# Diagnosis of hemogobinopathies in the clinical laboratory: an occult Hofu hemoglobin on HPLC

**DOI:** 10.1515/almed-2024-0175

**Published:** 2025-01-23

**Authors:** Maitane Echeverría Urroz, Ana Isabel López Delgado, Raquel Oliveros Conejero, David Álvarez Nistal

**Affiliations:** Laboratory of Biochemistry, Donostia University Hospital, Hernani (Gipuzkoa), Donostia, Spain

**Keywords:** hemoglobin Hofu, hemoglobinopaty, red blood cell series

## Abstract

**Objectives:**

Hemoglobinopathies are disorders affecting the structure, function and/or production of hemoglobin. These conditions are caused by mutations in the genes encoding globin synthesis. The highly variable clinical manifestations of hemoglobin disorders range from asymptomatic forms to severe anemia. Laboratory tests are crucial for diagnosis.

**Case presentation:**

We report the case of a patient who presented with asthenia. Since the patient had a family history of hemoglobonipathies, screening for erythropathies was performed. High-resolution liquid chromatography (HPLC) showed a normal distribution of hemoglobin levels. In contrast, capillary zone electrophoresis at alkaline pH demonstrated an unidentified rapid migration peak. Genetic testing revealed a mutation in the *HBB* gene causing Hofu hemoglobin disease.

**Conclusions:**

The hemoglobin variant Hofu is slightly unstable. While heterozygous carriers most frequently remain asymptomatic, they may develop anemia in the presence of other concomitant disorders. Distinctively, the retention time of Hb Hofu on HPLC is very close to that of HbA (0) and they often elute together. Therefore, Hb Hofu may remain masked, thereby leading to the misinterpretation of test results.

## Introduction

Hemoglobinopathies are a group of hemoglobin abnormalities with multiple manifestations. Laboratory testing is crucial for a diagnostic approach. A variety of methods are available in the clinical laboratory for the separation, quantification and detection of hemoglobin variants and thalassemias. We report a case of a rare hemoglobin variant, hemoglobin Hofu.

## Case presentation

We present the case of a 45-year-old woman from the north of the Iberian Peninsula with long-standing asthenia on follow-up in Primary Care (PC). As a history of interest, the patient had a maternal and paternal familial history of thalassemia of unknown etiology.

We received a request from PC including a basic complete blood count (CBC), biochemistry, HbA_2_ and HbF due to a familial history of thalassemia. Test results revealed microcytic iron deficiency anemia (Table 1). Upon suspicion that iron deficiency anemia was caused by an inherited erythropathy, screening for erythropathies was performed. We informed the requesting physician that iron deficiency should be corrected before testing, as iron deficiency may reduce HbA_2_ levels. After treatment was administered and iron deficiency was corrected, a complete hemogram was requested including a sample of serum (BD Vacutainer SST II Advance) and plasma (BD Vacutainer K_2_E (EDTA)). CBC demonstrated that hemoglobin levels had increased to reach the lower limit, along with microcytosis and a MCH concentration below the limit (Table 1). Hemolysis parameters (LDH, haptoglobin, total bilirubin and reticulocytes) were within normal limits.

**Table 1:** Anemia profile test results before and after oral iron supplementation to correct iron deficiency. Control laboratory testing was performed at 11 months.

Test, units	Baseline levels	After iron supplementation	Reference range
Hemoglobin, g/dL	9.1	12	12–15.3
Erythrocytes, µL	4.40 × 10^6^	4.88 × 10^6^	3.8–5 × 10^6^
MCV, fL	73	79.9	80–97
RCDW, %	17.2	19.5	11.5–15.6
MCH, pg	20.7	24.6	27–33
MCHC, g/dL	28.3	30.8	32–36
Iron, µg/dL	21	32	30–160
Ferritin, ng/mL	7	20	15–150

**Figure 1:**
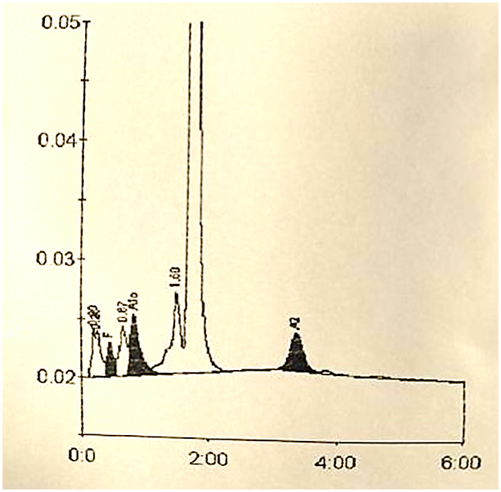
HPLC chromatography (BIO-RAD, D-10) coupled with photometric detection. Results are expressed in percentages and retention times: HbA: 82.3 %. 1.73 min; HbA_2_: 3.4 %. 3.36 min; HbF: 1.4 %. 0.46 min; HbAc: 5.1 %. 0.84 min.

**Figure 2:**
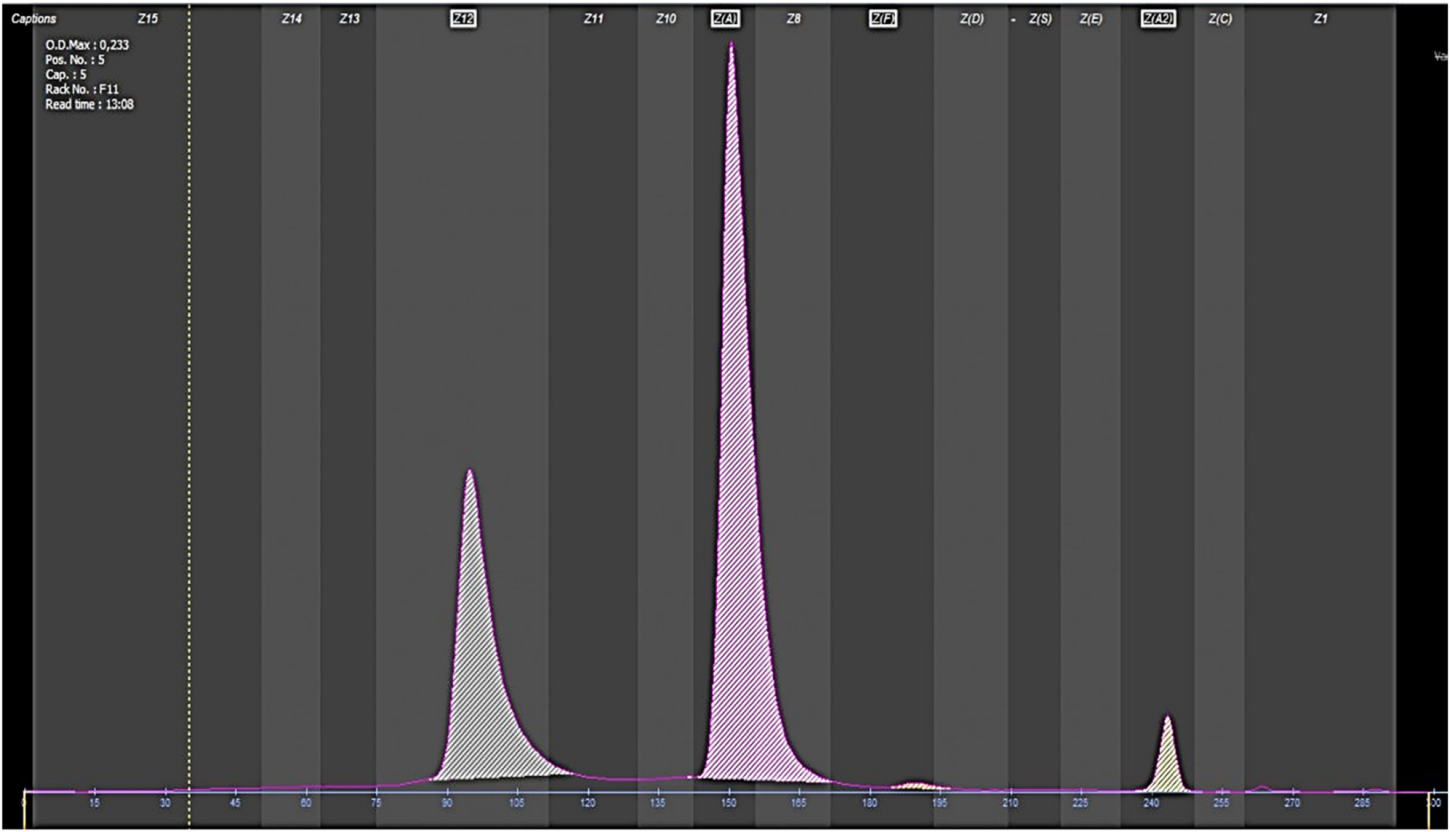
Capillary electrophoresis graph (Capillarys 2, Sebia) demonstrating peaks*.* From left to right, the grey peak indicates an unidentified peak, 31.9 %. The pink peak on the right represents HbA, 64.2 %. Next to the HbA peak, there is a small orange peak that represents HbF. On the right side, there is a yellow peak where HbA_2_ migrates, 3.4 %.

Screening for erythropathies was performed using two methods to detect potential occult hemoglobins by any of the two methods. The high-resolution liquid chromatography (HPLC) chromatogram (BIO-RAD, D-10) (Figure 1) excluded the presence of abnormal hemoglobin variants. Conversely, capillary electrophoresis (Sebia, Capillarys 2) revealed a peak of a fast-moving hemoglobin (Figure 2).

In the light of the growing suspicion of a hemoglobinopathy, genetic testing was recommended and the patient was referred to the Unit of Hematology. Previous hemograms were examined, showing episodes of iron deficiency anemia and reactive thrombocytosis without elevations in other series or associated cytopenias. Finally, testing was completed with Sanger sequencing of the alpha and beta globin genes.

Genetic testing revealed a mutation in the *HBB* gene in heterozygosis. This mutation has been previously described as the cause of hemoglobin Hofu [1].

Finally, the patient was diagnosed with Hofu hemoglobinopathy in heterozygosis. Follow-up by the Unit of Hematology involving regular hemograms and control visits were initiated, with the patient having remained asymptomatic.

Family screening is pending.

## Discussion

Hemoglobin consists of four globin chains, two alpha and two beta chains, along with four hemo groups. Different combinations of the alpha and beta chains result in different types of hemoglobins with distinct oxygen affinity and cooperative binding. This process is regulated genetically [2].

Hemoglobinopathies are a group of hereditary hematological disorders affecting globin chains. These are the most prevalent monogenic diseases. Hemoglobin disorders may affect the number, structure or function of one or several globin chains.

It is estimated that 7 % of the world population is carrier of a mutated gene. Between 300,000 and 500,000 affected infants are born annually with severe homozygous hemoglobin diseases, with thalassemia and sickle cell diseases being the most frequent [3].

Currently, migratory movements have led to an increase in the incidence of these disorders worldwide. As a result, hemoglobin diseases have become an emerging global health problem, with a growing number of cases in non-endemic regions.

The genetic mutation causing hemoglobin diseases may occur either in the genes encoding globin chains or at their regulatory regions, resulting in nonsense mutations, copy number variants, and gene fusions, among others. Therefore, it is a heterogeneous group of diseases. They can be categorized into two large groups: hemoglobin variants and thalassemias. This paper places the focus on hemoglobin variants, which contain a structural and, occasionally, a functional defect.

Hemoglobin disorders caused by hemoglobin variants arise from an amino acid change in the peptide chain [4]. An amino acid change may induce abnormalities in the physical and chemical properties of hemoglobin, thereby causing alterations in the structure, solubility, oxygen binding and physiological function of hemoglobin.

The most frequent hemoglobinopathy caused by a hemoglobin variant is the one resulting from HbS (sickle cell anemia). Nevertheless, more than 1,200 hemoglobin variants have been described in the literature.

The techniques available for detecting hemoglobin disorders include ion exchange HPLC, alkaline and acid electrophoresis, isoelectric focusing, capillary electrophoresis and molecular biology methods. Upon suspicion of a significant hemoglobin variant, international guidelines recommend using an appropriate alternative method for confirmation (level of evidence 1A) [5].

In the Laboratory of Clinical Chemistry of Donostia University Hospital, testing for hemoglobin disorders includes two techniques performed in parallel that are not mutually exclusive: Ion exchange HPLC and capillary zone electrophoresis at alkaline pH. This protocol facilitates the detection of potential hemoglobin variants masked by other peaks on the chromatography and vice versa.

Cation exchange HPLC is performed on a D-10 (Bio-Rad) system. The different hemoglobins are separated based on their ionic interaction with the cartridge material by a buffer gradient of increasing ionic strength. The separated hemoglobin fractions pass through the flow cell of the spectrophotometer, where absorbance is measured at a wavelength of 415 nm Absorbance is proportional to the hemoglobin fraction concentration and elutes at a specific time that is distinctive of the hemoglobin under study.

In turn, capillary electrophoresis is performed on a Capyllarys 2 (Sebia) system. The different hemoglobin fractions migrate based on their electrophoretic mobility through an electro-osmotic flow in an alkaline buffer. In alkalin pH, hemoglobin assumes a negative charge and migrates toward the anode (+) [6]. The different variants will reach the detector at separate time points based on their electrophoretic mobility.

The Hofu hemoglobin was first described by Miyaji et al. in Japan, who performed a study to identify hemoglobin variants in the population of the city of Hofu. During the study, the authors found a hemoglobin variant that migrated differently to HbA at alkaline pH, but that could not be differentiated from HbA at neutral pH. The new hemoglobin variant moved toward the agarose gel anode on electrophoresis at a pH of 8.6 [7], being a fast-moving hemoglobin. Genetic testing revealed a valine to glutamic acid substitution at position 126 of the β-globin chain [7].

Depending on the method used, the Hofu hemoglobin may not correctly separate from HbA, as it occurs in ion exchange HPLC chromatography, where separation is only partial. In contrast, in electrophoresis at alkalin pH, as migration to the anode is faster, the different hemoglobin fractions separate correctly [8].

In general, heterozygous carriers of the Hofu Hb variant remain asymptomatic or develop mild anemia, as it is a slightly unstable hemoglobin [8]. It is a rare hemoglobin disorder that has been reported in patients from Japan, Spain, Sri Lanka, India and America. It has also been described as concomitant to HbS and beta thalassemia [8], causing hemolysis when it occurs concurrently to the latter. When associated with HbS, patients are generally asymptomatic. Clinical manifestations include severe pain crises [9].

In our case, the patient had asthenia and mild anemia that was partially corrected with supplementation. Screening for erythropathies revealed the presence of an abnormal hemoglobin at 31 % by capillary zone electrophoresis at alkaline pH that could not be separated by cation exchange chromatography. According to the literature, the percentage of this hemoglobin in the absence of thalassemia range from 28 to 31 % [8]. Genetic testing excluded the presence of associated thalassemia and abnormal alpha chains. The hemoglobin variant was identified as Hofu Hb. The patient is on follow-up by the Service of Hematology.

## Lessons learned

– The clinical manifestations of hemoglobin variants are heterogeneous and heterozygous carriers usually remain asymptomatic. Diagnosis is essential, as homozygous forms or association with other hemoglobin disorders may lead to more severe forms.
– It is important that screening for potential hemoglobin variants includes two techniques, as this variant may be masked by other peaks.
– The hemoglobin Hofu is a rare structural hemoglobinopathy caused by a mutation in the *HBB* gene. Heterozygous carriers of this variant remain asymptomatic.
